# Traction apophysitis of medial malleolus: A case report with review of the literature

**DOI:** 10.4103/0019-5413.38589

**Published:** 2008

**Authors:** Rajiv Gupta, Sumit Batra, Ashu Verma, VK Sharma, Shabnam B Grover

**Affiliations:** Department of Orthopedics, Safdarjung Hospital, New Delhi,India; Vardhaman Mahavir Medical College and Associated Safdarjung Hospital, New Delhi, India; * Department of Radiodiagnosis, Safdarjung Hospital, New Delhi, India; Vardhaman Mahavir Medical College and Associated Safdarjung Hospital, New Delhi, India

**Keywords:** Apophysitis, malleolus

## Abstract

Traction apophysitis of medial malleolus is very rare and presented in view of its rarity. A 13 years old boy presented with pain and swelling without history of trauma around left ankle of 3 months duration. The swelling was diffuse with tenderness on anterior aspect of medial malleolus. The overlying skin was normal. The X-rays revealed fragmented accessory ossification centre of medial malleolus an left side. MRI revealed multiple foci of hypointensity in T1 and T2 weighted images of left medial malleolus apophysis. Patient was treated in below knee plaster for three weeks with restriction of sports activities for 5 weeks. The patient became asymptomatic in 8 weeks.

## INTRODUCTION

Traction apophysitis is a well-known entity and commonly seen in children involved in sports activities. Accessory ossification centers may appear as normal variant at the medial malleolus in growing children but traction apophysitis is very rare. We are reporting a case of traction apophysitis of medial malleolus in a child with review of the literature.

## CASE REPORT

**Figure 1:**
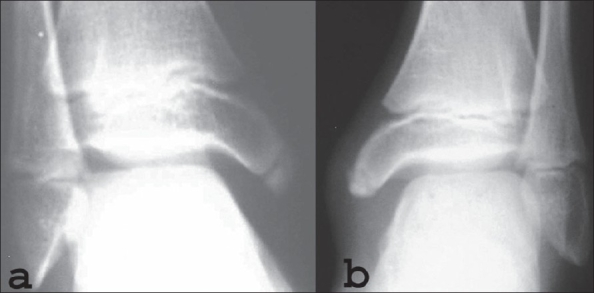
Anteroposterior X-ray of right (a) asymptomatic medial malleolus shows an accessory center. (b) Anteroposterior X-ray of left symptomatic (b) side shows soft tissue shadow and fragmentation of the accessory center

**Figure 2:**
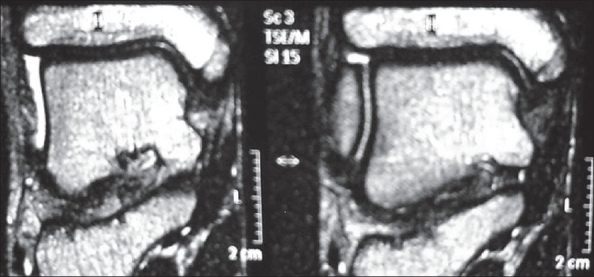
T2 WI MRI scan (coronal image) of the right ankle shows homogenous intensity of the accessory center

**Figure 3:**
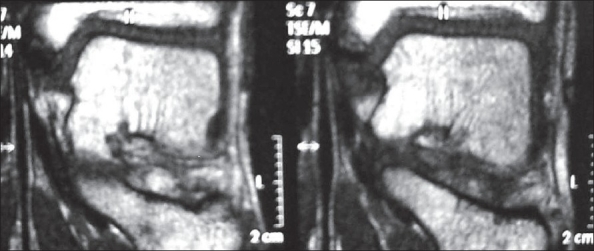
T2 WI MRI scan (coronal image) of the left side shows multifoci of hypointensity and fragmentation of the accessory center

A 13-year-old boy presented with pain and swelling around the left ankle region for three months. He was very active in outdoor sports activities. The pain used to increase on activity. There was no history of trauma. On examination there was diffuse swelling around the medial malleolus on the left side. Overlying skin was normal in appearance. Tenderness was present over the anterior aspect of the medial malleolus and there was pain on valgus stress applied to the ankle. Routine blood investigations were within normal limits. Plain X-ray of ankle showed accessory ossification center of the medial malleolus on both sides. The accessory ossification center on the right side showed uniform density with smooth margins and on the left side was fragmented [Figure 1]. An MRI scan was done for both the ankles. T1and T2 weighted coronal images showed homogenous signal intensity on the right side [Figure 2]. On the left side the apophysis showed multi-foci of hypo-intensity in both T1 and T2 weighted images. In addition the left medial malleolus apophysis appeared fragmented in both T1 and T2 weighted sequences [Figure 3].

On the basis of clinical and radiological findings a diagnosis of traction apophysitis of the medial malleolus was made. Below the knee POP slab was applied on the left side for three weeks and sports activities were restricted for another five weeks. The patient became asymptomatic in eight weeks.

## DISCUSSION

The traction apophyses are sites of active growth in children consisting of columns of growth cartilage uniting tendon with bone. Injuries at the traction apophysis may result from a single episode of macro trauma with resultant frank avulsion of a portion of apophysis or may result from repetitive micro trauma with resulting pain, swelling and on occasion bony and cartilaginous overgrowth referred to as apophysitis.

Another factor that is active in the occurrence of traction apophysitis is the growth process itself. Longitudinal growth occurs in the bones of the extremities and spine, with the soft tissue- muscle tendon units, ligaments and so on secondarily elongating in response to this growth. During this rapid growth, there can be a measurable increase in muscle-tendon tightness around the joints, with loss of flexibility and an enhanced environment for overuse injury. As the tight muscle tendon unit is subject to repetitive overload, there is an increased potential for repetitive tiny avulsion fractures at the weakest site in the muscle- tendon unit, the apophyseal growth cartilage.

The final factor associated with the onset of most of the traction apophysitis is repetitive stress applied to the apophysis, often in the form of repetitive sports training or competition.[1]

The accessory centers of ossification of the medial malleolus are common in skeletally immature individuals.[2–5] They usually appear between the ages of seven to 10 years and eventually fuse with the secondary ossification center of the malleolus at skeletal maturation. In a study of 100 normal children aged between six to12 years the accessory ossification center was found in the medial malleolus in 20%. In 13% it was present bilaterally.[2]

Many of the ossification centers are identified accidentally when radiographs are taken to evaluate injury at the ankle or foot. At times they may be mistaken for fractures. A smooth appearance on both sides of the radiolucency usually obviates the diagnosis.[5]

There have been very few reports describing symptomatic accessory center at the medial malleolus with no history of trauma.[2,4,5] Ogden and Lee attributed these symptoms to a cartilaginous fracture, fibrous union or pseudoarthrosis. They showed that bone scintigraphy was useful in the diagnosis.[5] Ishi, Miyagawa and Hayashi[4] showed that MRI was useful in demonstrating the pathological changes of traction apophysitis of the medial malleolus, which cannot be demonstrated by radiography or scintigraphy. They attributed the cause of traction apophysitis to increased mechanical stress at the anterior part of the malleolus, plus increasing developmental weakness at the same site. During development, the deltoid ligament of the ankle is relatively shortened by the bone growth and stressed by repetitive ankle eversion during sports activities. Traction forces are concentrated on the anterior part because of the fan-like shape of the tibionavicular and tibiocalcaneal parts of the ligament, which are attached to it. Hyperpronation of the feet may increase traction forces in the deltoid ligament.[4]

Conservative management in the form of restriction of activities with or without immobilization leads to resolution of symptoms in one to six months depending on the severity of injury.[4,5]

The case described in this report showed fragmentation of the accessory center on both the X-ray and MRI. Clinico-radiological diagnosis of apophysitis of the medial malleolus was made and conservative treatment was given in the form of plaster slab and rest which showed good results.

## CONCLUSION

Extra centers of ossification at the tip of the internal malleolus are common in children. Most of them remain asymptomatic and eventually fuse with the lower tibial epiphysis. A few of them become symptomatic in children involved in sports activities due to repetitive trauma. Conservative management in the form of restriction of activities and splintage gives good results.
